# CaFap–Alumina Composite Bioceramics Based on the New CaAlO_2_F Phase

**DOI:** 10.3390/ma19194116

**Published:** 2026-09-26

**Authors:** Adriana Barylyak, Viktor Zinchenko, Stanisław Adamiak, Svitlana Pavlinchuk, Grygoriy Dmytriv, Volodymyr Pavlyuk, Yaroslav Bobitski, Małgorzata Sznajder

**Affiliations:** 1 Department of Therapeutic Dentistry, Danylo Halytsky Lviv National Medical University, Pekarska 69b, 79010 Lviv, Ukraine; 2 Department of Chemistry of Functional Inorganic Materials, Bogatsky Physico-Chemical Institute of the National Academy of Sciences of Ukraine, Lustdorfska Doroga 86, 65080 Odessa, Ukraine; 3 Institute of Materials Engineering, University of Rzeszow, Pigonia 1, 35-959 Rzeszow, Poland; sadamiak@ur.edu.pl; 4 Department of Inorganic Chemistry, Ivan Franko Lviv National University, Kyryla i Mefodia 6, 79005 Lviv, Ukraine; volodymyr.pavlyuk@lnu.edu.ua; 5 Institute of Physics, University of Rzeszow, Pigonia 1, 35-959 Rzeszow, Poland; ybobytskyy@ur.edu.pl

**Keywords:** apatite-alumina composites, fluorapatite, porous ceramics, bioceramics, sintering

## Abstract

We report on the synthesis of a new CaAlO_2_F phase with a perovskite-type structure, which was formed as a third phase in the CaFap–alumina composite during the calcination of a mixture of Ca_10_(PO_4_)_6_F_2_ and Al(OH)_3_. The atomic coordinates, lattice constant, and space symmetry group of the CaAlO_2_F phase were determined by the XRD method. Next, the chemical composition and mechanical properties of the CaFap–alumina composite bioceramics were investigated. The material was found to exhibit high porosity, with pore diameters ranging from 100 to 1000 μm. The measured mean values of microhardness and Young’s modulus were 6.1 ± 1.0 GPa and 103 ± 8 GPa, respectively. The new CaAlO_2_F phase constituting the bioceramics exhibited a significantly higher nanohardness of 11.8 ± 1.5 GPa and Young’s modulus of 137 ± 9 GPa, which indicates that this phase can significantly improve the mechanical properties of CaFap–alumina bioceramics. The resulting fluorapatite-based biocomposite can pave the way for advanced medical ceramics as a material for load-bearing implants.

## 1. Introduction

Developing suitable materials for prostheses and implants used in cases of bone or dental tissue damage has long been a significant challenge [1]. There are various types of materials which are applied for these purposes: (i) inactive materials (some metals, e.g., Ti, Ta), (ii) bio-inert materials (oxides, particularly ZrO_2_ and Al_2_O_3_), and (iii) bioactive materials (apatites, e.g., hydroxyapatite Ca_10_(PO_4_)_6_(OH)_2_ (CaHap) or fluorapatite Ca_10_(PO_4_)_6_F_2_ (CaFap), tricalcium phosphate Ca_3_(PO_4_)_2_ or mixed phosphate-silicate glasses, other phosphates).

While titanium and its alloys are widely used in implantology, calcium phosphate bioceramics are the most widely used materials in bone and maxillofacial surgery due to their chemical similarity to bone tissue [2]. For implantology applications, the most promising candidates are composites or coatings in which a bioactive material (e.g., CaHap or CaFap) is combined with an inactive material (titanium and its alloys) or a bioinert material (oxides) to produce a bioactive implant with high mechanical strength and durability. Calcium apatites are by far the most common materials within the bioactive group. Highly porous, apatite-based bioceramic scaffolds have been extensively studied as three-dimensional (3D) templates for cell adhesion, proliferation, and differentiation, promoting bone regeneration [3,4]. However, their brittleness limits their clinical application, particularly in cases of large bone defects. This is especially true for hydroxyapatite (CaHap), which is not an ideal solution. Furthermore, CaHap has exhibited certain shortcomings regarding its mechanical properties [5]. Additionally, the degradation rate of CaHap coatings in a biological environment represents a significant drawback [6].

Recently, CaFap has attracted attention as an alternative to pure CaHap coatings on metal implants, again due to the similarity of its chemical composition to bone minerals. In fact, CaFap offers a potential advantage over CaHap thanks to its superior chemical stability and ability to inhibit the caries process without compromising biocompatibility [7]. Human bone is known to contain approximately 1 wt.% fluorine [8]; the fluoride ion itself protects teeth against caries and enhances stability, even at elevated temperatures. The fluorine present in CaFap plays a decisive role in shaping the material’s physical and biological properties [9].

We recently published a comprehensive comparative study on hydroxyapatite and fluorapatite as bases for producing bone scaffolds [10]. The results demonstrated that CaHap- and CaFap-based bone scaffolds exhibit a macroporous microstructure with an interconnected pore network and compressive strength comparable to that of cancellous bone. These biomaterials are biodegradable under both physiological (pH 7.4) and acidic (pH 4.5) conditions, mimicking the osteoclast-mediated bone resorption process. Furthermore, they display high biocompatibility and osteoconductive properties, as well as the ability to induce apatite formation on their surfaces, indicating bioactivity. Moreover, CaHap- and CaFap-based scaffolds support osteogenic differentiation and do not trigger excessive ROS/RNS production by immune cells, thereby enhancing their potential for biomedical applications. We also showed that CaFap is the superior bioceramic component for producing this specific type of bone scaffold, owing to the higher porosity and inherent bioabsorbability of the CaFap-based material compared to the CaHap-based counterpart [10]. This is a crucial property from a clinical perspective, as it facilitates the gradual replacement of the scaffold with newly formed bone tissue.

The incorporation of ceramic oxides is also being considered a potential option for reinforcing such bone scaffolds [11,12,13]. Among bioinert ceramic reinforcements, aluminum oxide (Al_2_O_3_) is successfully used in orthopedic applications due to its excellent wear resistance [1]. There are also numerous studies concerning the sintering processes and mechanical properties of Al_2_O_3_-CaHap-based composites [14,15,16]. However, only a limited number of studies address the sintering of Al_2_O_3_-CaFap-based composites; thus, research into the synthesis and coating technology of CaFap–aluminum oxide composites as alternative solutions is also important [17,18,19]. The authors of [20] evaluated the effect of an Al_2_O_3_-CaFap composite coating applied to 316L stainless steel on integration with rabbit bone, focusing on biocompatibility and bioactivity (in vitro and in vivo studies). The authors demonstrated that implants coated solely with aluminum oxide showed no signs of integration with bone tissue. By contrast, the Al_2_O_3_–Fap coating performed excellently in the tests, proving that the Al_2_O_3_–Fap-based composite is bioactive and effectively improves biocompatibility at the bone–implant interface.

The positive results of a series of studies on the aforementioned composites provide strong motivation to search for technologies and synthesis methods, not only for composite surface coatings for implants, but also for producing bulk materials for bone surgery and tissue engineering. The aim of the present study is to develop a low-cost synthesis method for a new multiphase composite based on fluoroapatite (CaFap) and alumina (Al_2_O_3_), and to characterize its key structural and physicochemical properties.

## 2. Materials and Methods

Studies on the synthesis of a new composite including both CaFap and alumina (Al_2_O_3_) were carried out. Immediately prior to these studies, we developed an essentially new way of synthesizing CaFap in a saline melt (NaCl-KCl) with NaCl (99.99%, Thermo Scientific Chemicals, Waltham, MA, USA) and KCl (99.99%, Sigma-Aldrich Chemie GmbH, Taufkirchen, Germany) at a moderate temperature (700 °C). For the synthesis of CaFap, CaCO_3_ (99.995%, Sigma-Aldrich Chemie GmbH, Taufkirchen, Germany), CaO (99.99%, Sigma-Aldrich Chemie GmbH, Taufkirchen, Germany), CaF_2_ (99.99%, Thermo Scientific Chemicals, Waltham, MA, USA) and NaPO_3_ (99.99%, ATT Elements, Albany, NY, USA) were used as initial components containing Ca and P. The reaction scheme can be described by

(1) $${3\mathrm{CaCO}}_{3}+6\mathrm{CaO}+{\mathrm{CaF}}_{2}+{6\mathrm{NaPO}}_{3}\;\overset{T,\;\mathrm{Ar}}{\to }\;{\mathrm{Ca}}_{10}{\left({\mathrm{PO}}_{10}\right)}_{6}F_{2}+{3\mathrm{Na}}_{2}{\mathrm{CO}}_{3}.$$

To prevent potential hydrolysis, it was preferable to carry out the process under an inert gas (argon) atmosphere. The XRD data suggested the formation of a well-crystallized product. Water-washed powders contained a large quantity of nanoscale particles bound into stick-like aggregates of various sizes. Next, in order to obtain a composite of CaFap and alumina (Al_2_O_3_) in a weight ratio of 2:1, Ca_10_(PO_4_)_6_F_2_ and Al(OH)_3_ (99.99%, Aladdin Scientific, Shanghai, China) were used as starting components. The composite CaFap–alumina was synthesized in a solid-state phase. The starting mass (powder mixture) was pressed into pellets and sintered in a tube furnace (RHTC 80-450, Nabertherm, Lilienthal, Germany) in three stages for 2 h each: stage 1—650 °C; stage 2—1000 °C; and stage 3—up to 1350 °C. After each stage, the tablets were milled and pressed again. Thus, we obtained strong white tablets (with a light gray tint).

The structure of the CaFap-Al_2_O_3_ composite specimen was characterized by an X-ray powder diffraction pattern recorded on a STOE STADI P diffractometer (STOE & Cie GmbH, Darmstadt, Germany) (CuK_α_ radiation) in the range of 2*θ* = (6.000 − 110.295) degrees with a step of 0.015. The calcium fluorapatite model was adopted as the standard crystal structure model [21].

Next, metallographic examination was performed using a Nikon SMZ 745T optical microscope (Nikon, Tokio, Japan). Quantitative analysis of the structural components was performed using the Multiscan system. The structure and chemical composition of the composite sample were examined using an FEI Quanta 3D 200i SEM (FEI Company, Hillsboro, OR, USA). Chemical composition and elemental concentration profiles were obtained using an EDAX Ametek energy-dispersive spectrometer (EDS) (FEI Company, Hillsboro, OR, USA).

The mechanical properties of CaFap–alumina were tested using the indentation method, which involved measuring the material’s deformation by compressing an indenter with a fixed force. The indenter force and the residual depth of indentation in the material were recorded during a constant loading and unloading cycle. Based on the obtained load-depth indentation curves, the hardness and Young’s modulus values were determined. Both micro- and nanohardness measurements of the composite sample were performed using CSM hardness testers (CSM Instruments, Peseux, Switzerland). To obtain more representative results, microhardness measurements of the whole CaFap–alumina composite were carried out using higher loads. The microhardness was measured under a load of 100 mN using a Vickers indenter (CSM Instruments, Peseux, Switzerland). As is well known, due to local variations in material properties, point measurements performed at lower loads may characterize only a limited area of the composite and do not necessarily accurately reflect its overall hardness. Therefore, when determining the hardness of the individual phases of the CaFap–alumina composite, lower loads (nanohardness measurement) were applied to minimize the influence of the surrounding phases on the measurement result. The nanohardness of the sample was determined under a load of 10 mN using a Berkovich indenter (CSM Instruments, Peseux, Switzerland). In both cases, the dwell time at the maximum load was 15 s. A series of 10 measurements were performed to obtain the mean value of both micro- and nanohardness.

## 3. Results and Discussion

### 3.1. XRD Analysis

Figure 1 presents the differential diffractogram obtained after the refinement of the CaFap crystal structure by the Rietveld method using Full Prof program [22]. As can be seen, the specimen, besides CaFap, contains an additional phase with the basic peaks at 31.256, 38.525 and 44.792 degrees of 2*θ*. It was challenging to identify this additional phase as Al_2_O_3_ based on the initial components Ca_10_(PO_4_)_6_F_2_ and Al(OH)_3_ before sintering because none of the alumina phases are characterized by such a set of highest-intensity peaks. This is true for the thermodynamically stable α-Al_2_O_3_ (corundum structure type, $R\bar{3}c$ space group) [23] and several transitional or metastable phases: γ-Al_2_O_3_ (cubic spinel structure type, $\mathrm{Fd}\bar{3}m$ space group) [24], γ’-Al_2_O_3_ (Al_2_O_3_ structure type, $P\bar{4}m2$ space group) [24], κ-Al_2_O_3_ (Fe_2_O_3_ structure type, *Pna*2_1_ space group) [25], θ-Al_2_O_3_ (Ga_2_O_3_ structure type, *C*2/*m* space group) [26], η-Al_2_O_3_ (defect spinel structure type, *Fd*3*m* space group) [26], χ-Al_2_O_3_ (hexagonal structure, *P*6_3_*mc* space group) [27], Al_2_O_3_ (tetragonal structure, *I*4_1_/*amd* space group) [28], and δ-Al_2_O_3_ (Al_2.67_O_4_ structure type, $P\bar{4}m2$ space group) [29]. By automatic indexing of the basic peaks of the additional phase using the TREOR 90 program [30], we established that this phase crystallizes with cubic symmetry, with the lattice parameter *a* = 4.0436(1) Å.

Given that studies show that the high-pressure Al_2_O_3_ phase adopts the CaIrO_3_ post-perovskite-type structure [31], and considering that calcium titanate CaTiO_3_ undergoes two reversible phase transitions—from orthorhombic *Pbnm* to tetragonal *I*4/*mcm* and from tetragonal *I*4/*mcm* to cubic $\mathrm{Pm}\bar{3}m$ symmetry structures [32]—it is reasonable to assume that the additional phase under analysis may indeed possess a perovskite-type structure. The compound CaAl_0.5_Sb_0.5_O_3_ also exhibits this structure [33], where there is a statistical mixture of Al^3+^ and Sb^5+^ ions in a 1*a* Wyckoff position instead of Ti^4+^ in the perovskite structure, which allows the above-mentioned compound to maintain a neutral balance of electrons. Since our specimen contains Ca, Al, O and F atoms, it can be assumed that a chemical compound with a perovskite-type structure was formed, in which, in the 1*a* Wyckoff position, instead of Ti^4+^ ions, Al^3+^ ions are present (their ionic radii are similar and equal to 0.74 and 0.68 Å, respectively), and in the Wyckoff 3*d* position, to maintain charge neutrality of the compound, a statistical mixture of O^2−^ and F^−^ ions is present in a molar ratio of 2:1. Therefore, the crystal structure of the new CaAlO_2_F phase was refined in the CaTiO_3_-type structure (space group $\mathrm{Pm}\bar{3}m$) with a fixed molar ratio of O^2−^ and F^−^ ions. The atomic parameters of CaAlO_2_F are presented in Table 1 and the refined, two-phase powder pattern of the CaFap–alumina composite material is presented in Figure 2. The weight fractions of both phases are 81.0% of CaFap and 19.0% of CaAlO_2_F.

Since the initial components Ca_10_(PO_4_)_6_F_2_ and Al(OH)_3_ were used in a 2:1 mass ratio for the synthesis, we can predict that the remaining Al(OH)_3_ transformed into an amorphous Al_2_O_3_ phase. It is also worth noting that the peaks of the newly formed CaAlO_2_F phase are quite broad compared to those of the CaFap phase, suggesting a nanostructural nature of this new phase.

### 3.2. Morphology and Mechanical Properties of the CaFap–Alumina Composite

Since one of the reaction products is a gaseous substance (H_2_O), it can be assumed that the resulting new composite must have a porous structure. Actually, metallographic studies using a Nikon stereomicroscope with 5× magnification confirm this assumption (see Figure 3).

The porosity of the CaFap–alumina ceramic structure is calculated using the MultiScan quantitative analysis software (MultiScan v. 18.03). The resulting mean porosity is evaluated to be 27.9%, with pore sizes in the interval from 100 to 1000 μm (1 mm). The majority of pores range in size from 100 to 200 μm, as shown in the histogram (Figure 4).

At significantly higher magnification (1000×), it can be observed (Figure 5) that the CaFap–alumina ceramic material consists of three components: “light gray”, “gray”, and “dark gray”.

These components can be identified in the following way. The composite contains 81.0% CaFap, 19.0% CaAlO_2_F, and amorphous Al_2_O_3_. The interaction mechanism in the system under study is as follows. The starting components of the system are CaFap (66.67%) and Al(OH)_3_ (33.33%); hence, the reaction mechanism can be described by a schematic equation:

(2) $${\mathrm{Ca}}_{10}({\mathrm{PO}}_{4}{)}_{6}F_{2}\;+\;\mathrm{Al}(\mathrm{OH}{)}_{3}\;\overset{T,\;\mathrm{Ar}}{\to }\;{\mathrm{Ca}}_{10}(H_{3}O)({\mathrm{PO}}_{4}{)}_{6}F+{\mathrm{CaAlO}}_{2}F.$$

This process is the primary one. The excess Al(OH)_3_ present in this study decomposes according to

(3) $$2\mathrm{Al}{\left(\mathrm{OH}\right)}_{3}\overset{T,\;\mathrm{Ar}}{\to }\;{\mathrm{Al}}_{2}O_{3}+3H_{2}O↑.$$

CaFap reacts completely to form a substituted fluoroapatite; Al(OH)_3_ is partially consumed in reaction (2), while the remainder decomposes into H_2_O and amorphous Al_2_O_3_. Calculations based on stoichiometry indicate the formation of 11.38 g of the new phase and 55.29 g of the substituted CaFap phase, yielding a ratio of 4.86. The ratio determined via the Rietveld method is 81:19, or 4.26. In general, the Rietveld method does not account for the presence (at least in significant quantities) of an amorphous component such as Al_2_O_3_. To the best of our knowledge, no direct methods for quantifying the amorphous phase content have yet been developed; therefore, we rely on calculation-based methods. Indeed, metallographic analysis (based on crystallite surface area) indicates that the amorphous component (Al_2_O_3_) content should be 34.4 wt.%. At the same time, stoichiometric calculations yield a value of 29.4 wt.%, indicating that the results are close to one another. The intensity of the coloration (the grayness of the phases in Figure 5) can be explained as follows. The CaFap phase appears the lightest (“light gray”) due to the stable valence state of its constituent atoms. It is worth noting that the crystallites of this phase exhibit a hexagonal shape, also consistent with the CaFap hexagonal crystal structure. The Al_2_O_3_ component (amorphous)—which is dispersed throughout the volume rather than being incorporated into any of the phases—appears darker (“gray”). In this instance, the presence of cations with different charges (Al^3+^, Al^2+^) cannot be ruled out, as this imparts a gray color to the phase. Finally, the new compound CaAlO_2_F appears the darkest (“dark gray”); it likely contains anions with different charges (O^2−^, O^−^).

We independently determined the content of the “light” component in the investigated sample using the MultiScan program, finding a value of 39.6%; before this, we binarized the image.

A similar composition, i.e., a mixture of “light” (fluoroapatite) and “dark” (alumina) phases, was also observed by the authors [17] in a coating made of an Al_2_O_3_-Fap composite.

Hardness testing of the CaFap–alumina composite structure began with microhardness measurements using a Vickers indenter at a load of 100 mN, covering the area of all three phases, yielding a mean value of 6.1 ± 1.0 GPa. The amorphous Al_2_O_3_ phase certainly contributes significantly to this value; according to the literature, this phase exhibits a hardness in the 8–12 GPa range [34]. By contrast, for the nanohardness measurements of individual phases (at a load of 10 mN using a Berkovich indenter), the indentation sites were precisely selected—specifically, the “dark gray” and “light gray” phase regions of the sample. These two regions yielded distinct values: 11.8 ± 1.5 and 7.2 ± 0.6 GPa, respectively. Thus, it can be concluded that the individual contribution of the dark phase—specifically that attributed to CaAlO_2_F—to the overall hardness of the biocomposite is significant. The measured mean value of Young’s modulus was 103 ± 8 GPa, with values of 80 ± 10 GPa for the “light” phase and 137 ± 9 GPa for the “dark” phase (see Figure 6).

The obtained results indicate that the investigated multiphase ceramics may find application in two key areas: the bioactive coating of implants (e.g., based on CaHap-ZrO_2_ [35]) and as scaffolds in bone tissue engineering [36,37].

### 3.3. SEM/EDS Analysis

The microstructure revealed by SEM confirms the heterogeneous phase composition of the CaFap–alumina ceramics (see Figure 7).

The EDS analysis performed (Figure 8 and Figure 9) shows different contents of elements in various cross-sections along the gray test line shown in Figure 7. The position of the positive deviation in the fluorine spectral peak coincides with the negative deviation in the oxygen peak; this corresponds to the CaAlO_2_F phase, which exhibits the highest fluorine content (20 at.%) and the lowest oxygen content (40 at.%). For comparison, the fluorine content in CaFap is only about 2 at.%, while the oxygen content is 60 at.%. The absence of phosphorus in this phase is also noteworthy, as it is entirely consistent with the composition of the new phase.

A characteristic contrasting change in the content of Al and Ca, P, O, and F is particularly noticeable in the area of intersection of the narrow “light” strip near the center of the cross-section. This is understandable given the phases in which these elements are distributed: P—primarily in the CaFap phase; F—in the new CaAlO_2_F phase; and Al—in the amorphous Al_2_O_3_ component. The relatively high content of C, K, and especially Na turned out to be somewhat unexpected; apparently, these latter elements are remnants of incompletely washed NaCl and KCl, which formed the basis of the salt melt. However, the overall ratio of elements does not fully correspond to the composition of the phases forming the CaFap-Al_2_O_3_ apatite ceramics. It should be noted that the presence of NaCl and KCl in the sample does not adversely affect its properties; moreover, they accelerate the interaction and improve its biocompatibility.

## 4. Conclusions

We presented a method of synthesizing a CaFap–alumina composite in which a new CaAlO_2_F phase with a perovskite-type structure (space group $\mathrm{Pm}\bar{3}m$, lattice constant *a* = 4.0436(1) Å) was identified. This phase was formed during the calcination of a mixture of Ca_10_(PO_4_)_6_F_2_ and Al(OH)_3_, and appeared as a third component in the CaFap–alumina composite alongside the fluorapatite and amorphous aluminum oxide phases. The new CaAlO_2_F phase was identified based on X-ray diffraction (XRD) analysis, EDS chemical composition analysis, and micro- and nanohardness measurements of the resulting CaFap–alumina composite. The mechanical properties of the composite were investigated by determining its hardness and Young’s modulus. We demonstrated that the CaFap–alumina composite containing the new CaAlO_2_F phase exhibits a highly porous structure, with an average porosity of 27.9% and pore sizes ranging from 100 to 1000 μm. The majority of pores fall within the 100–200 μm range. This property is crucial for osteoblast adhesion and the ingrowth of blood vessels (angiogenesis) through the pores. The mean values of microhardness and Young’s modulus for the entire CaFap–alumina composite are 6.1 ± 1.0 and 103 ± 8 GPa, respectively. The new CaAlO_2_F phase within the composite exhibits a much higher nanohardness of 11.8 ± 1.5 GPa and a Young’s modulus of 137 ± 9 GPa.

Our research results thus present a technologically advanced solution to the primary challenge of bioceramics: combining biocompatibility with material strength. The well-known, excellent biological properties of fluoroapatite are enhanced by the exceptional mechanical strength and wear resistance of Al_2_O_3_, as well as the presence of a new phase. Consequently, the synthesized multiphase composite offers significant advantages over pure fluoroapatite. The next stage of our research will focus on controlling the bioactivity of the CaFap–alumina composite (specifically, fluoride release and associated toxicity risks) and investigating its application in two key areas: bioactive implant coatings and dental structures and fillings (crown elements, post-and-core systems, and cement components).

## Figures and Tables

**Figure 1:**
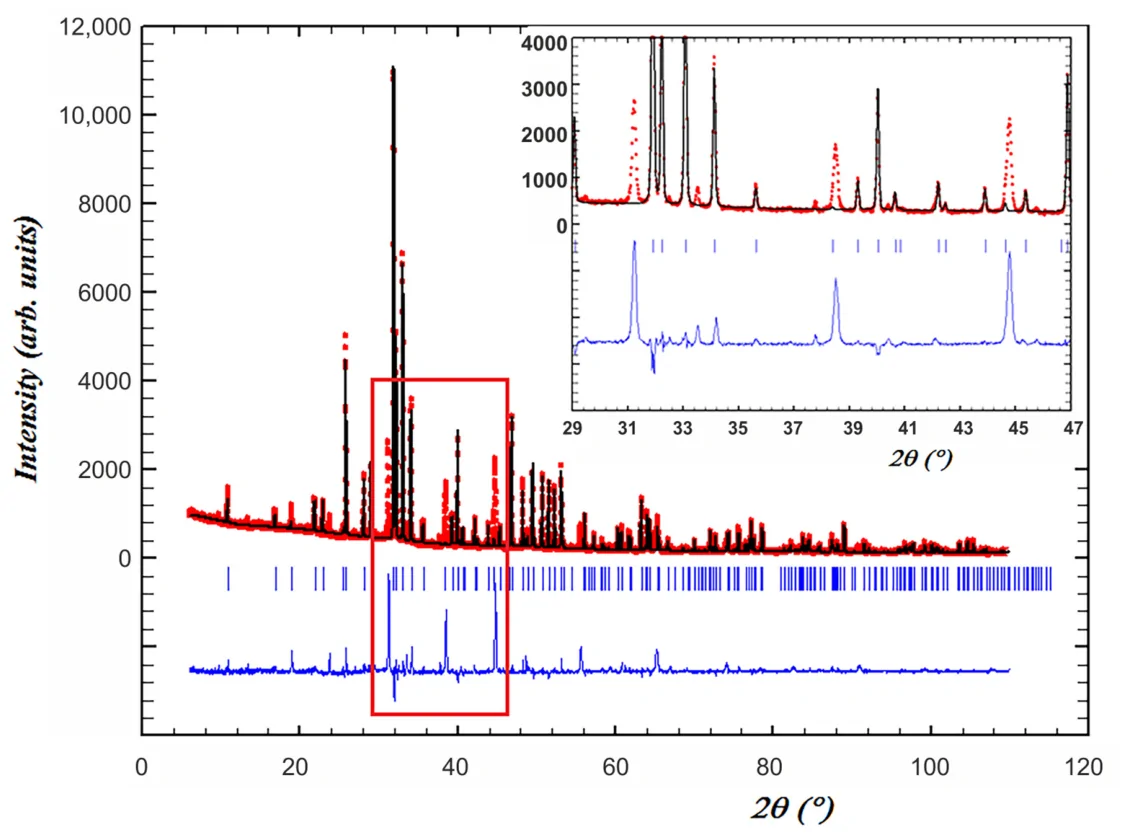
Observed (red), calculated (black line), and difference (bottom blue line) X-ray powder diffraction patterns of CaFap.

**Figure 2:**
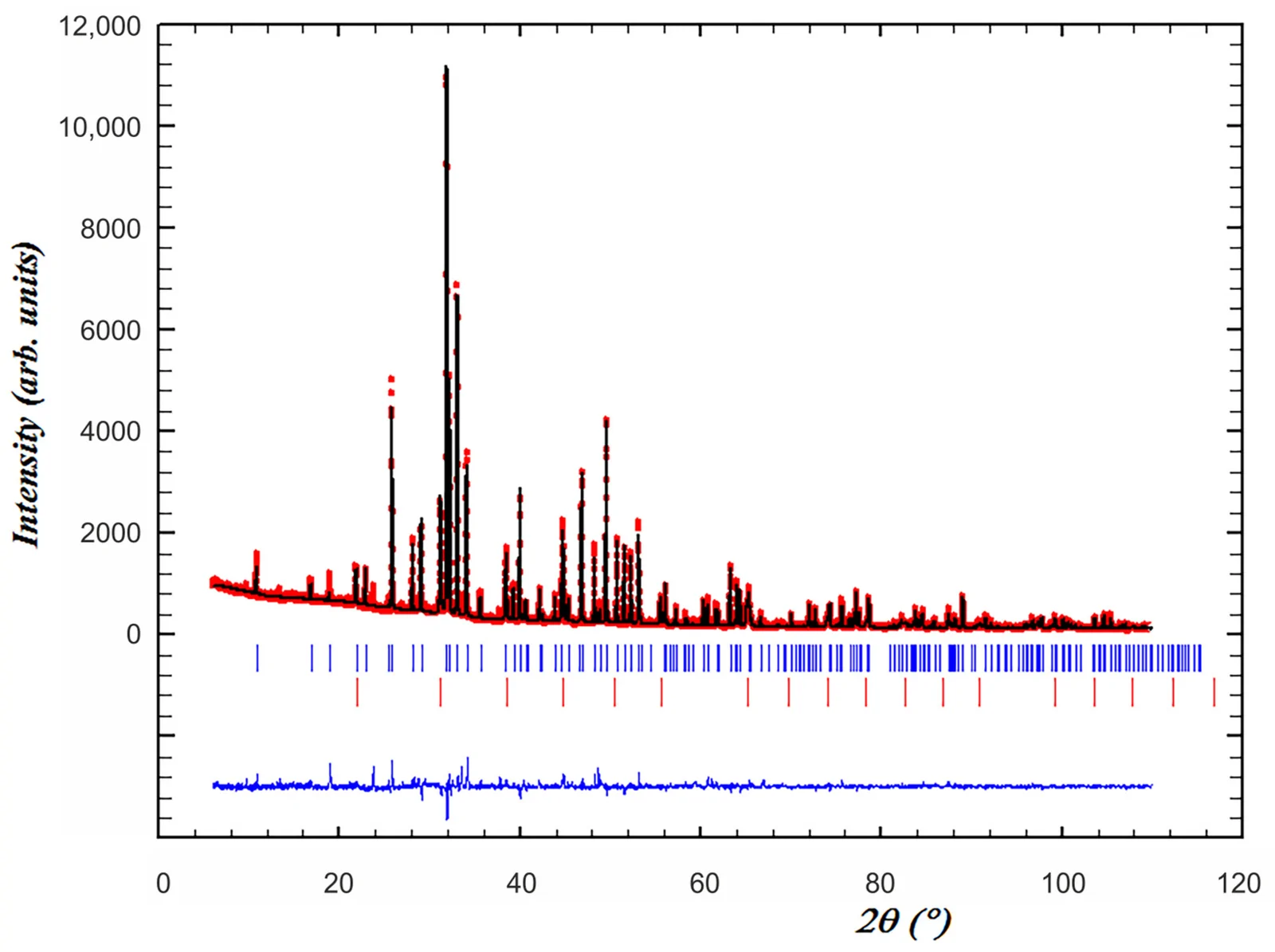
Observed (red), calculated (black line), and difference (bottom blue line) X-ray powder diffraction patterns of the composite material CaFap (blue bars of Bragg reflections) and CaAlO_2_F (red bars of Bragg reflections).

**Figure 3:**
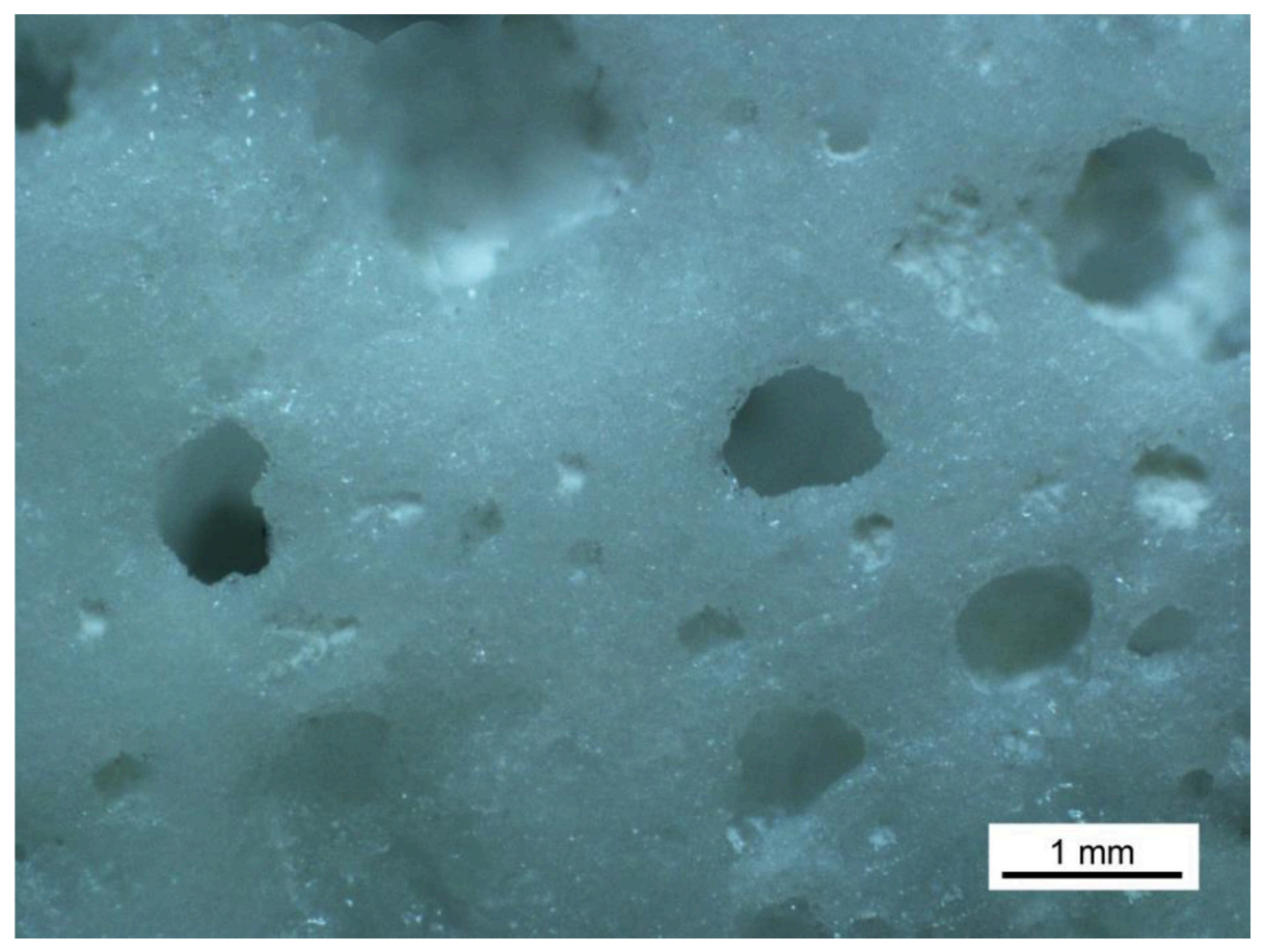
Structure of CaFap–alumina porous ceramics.

**Figure 4:**
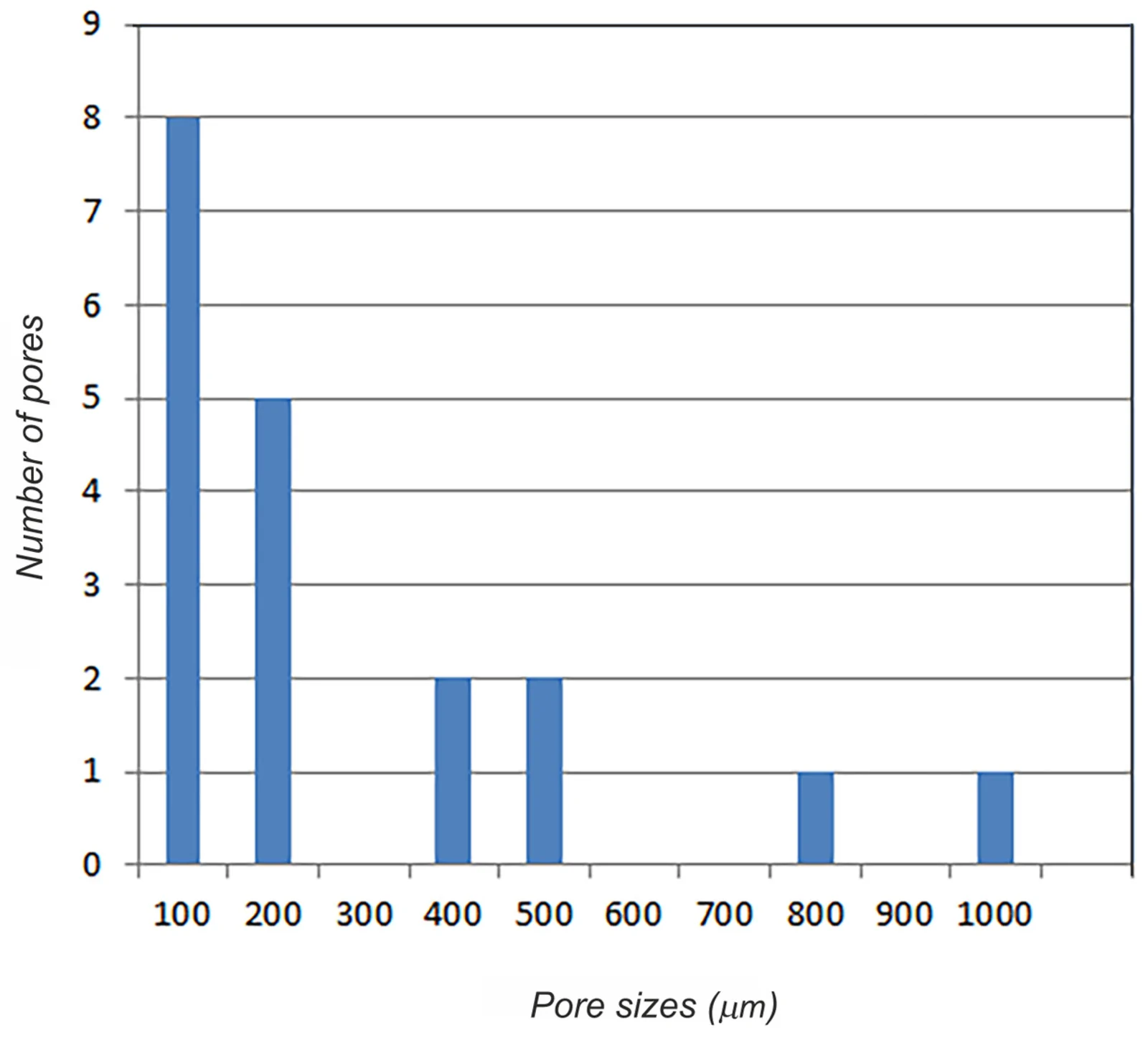
The distribution of the pore sizes in CaFap–alumina (surface of 2.35 × 3.15 mm^2^).

**Figure 5:**
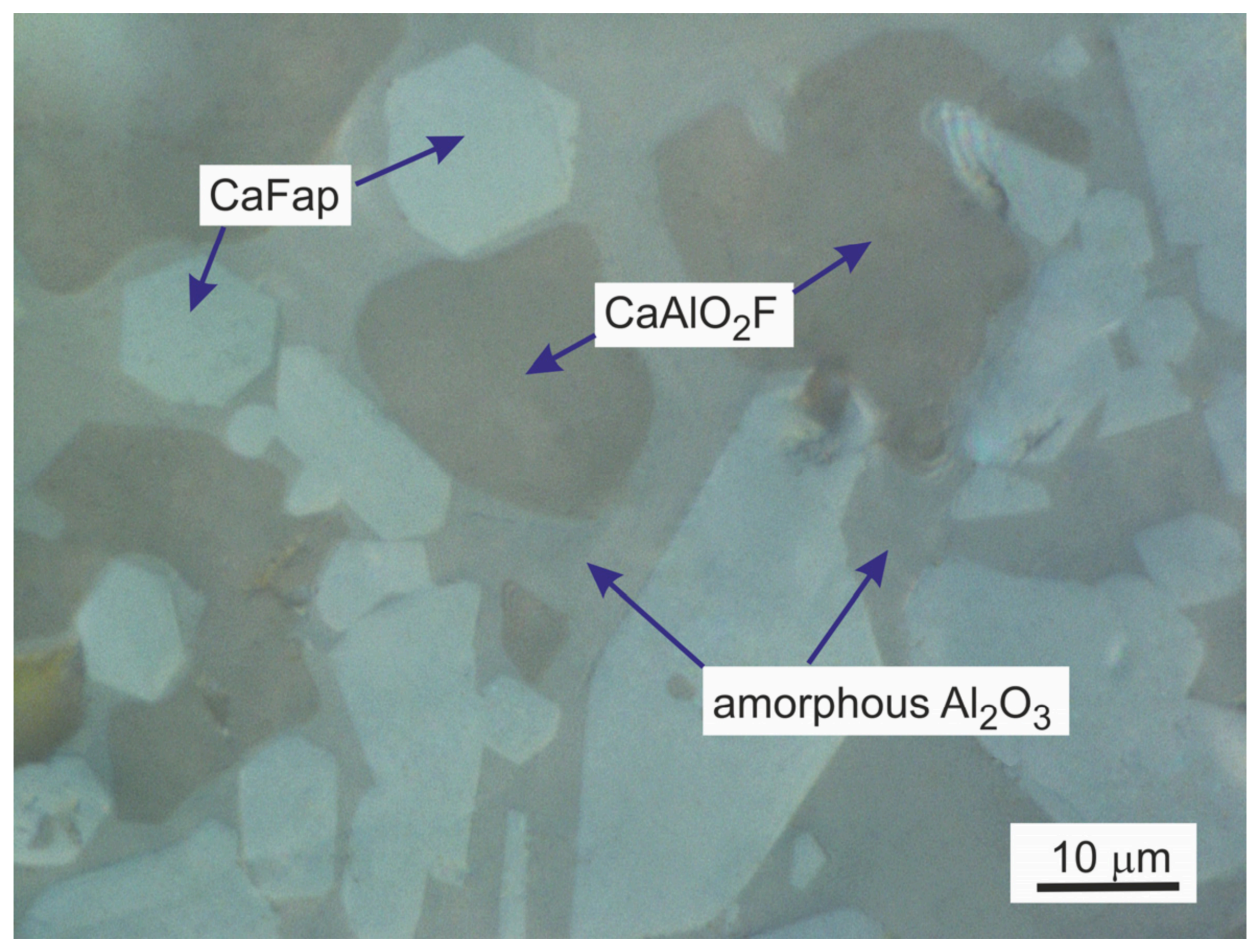
Surface structure of CaFap–alumina ceramics (1000× magnification).

**Figure 6:**
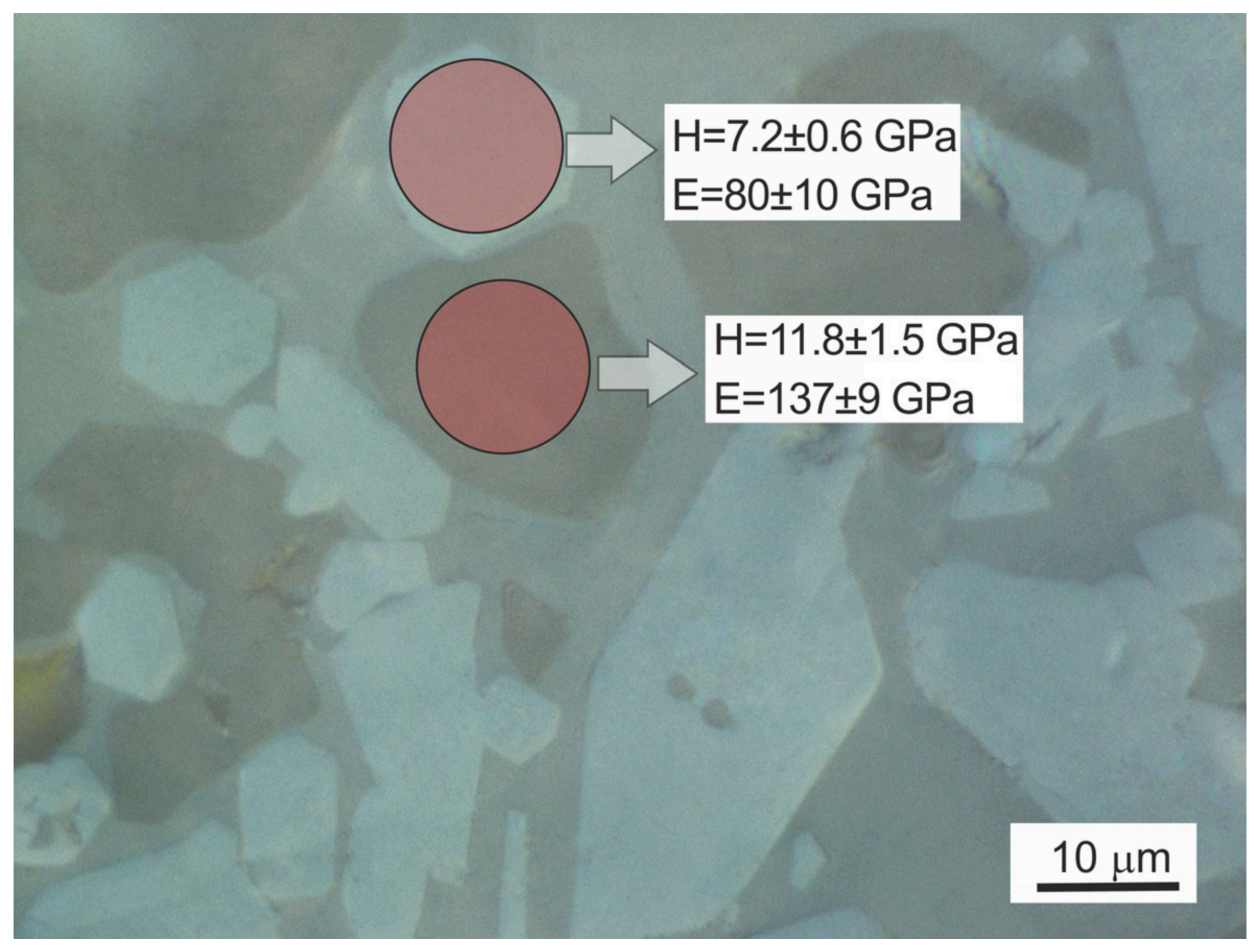
Nanohardness (top) and Young’s modulus (bottom) of CaFap–alumina porous ceramic phases.

**Figure 7:**
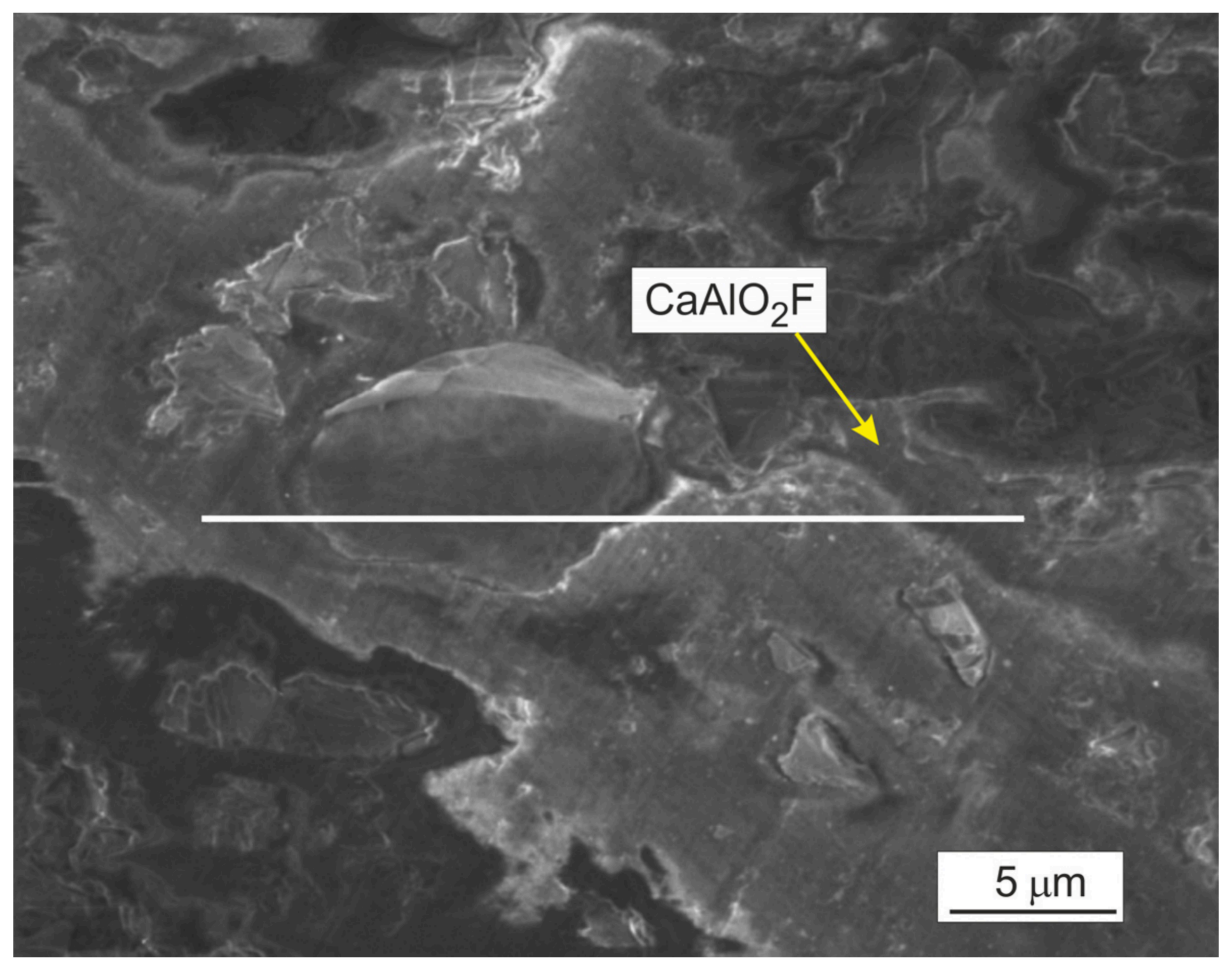
Multiphase microstructure (SEM) of CaFap–alumina ceramics with the EDS test line indicated.

**Figure 8:**
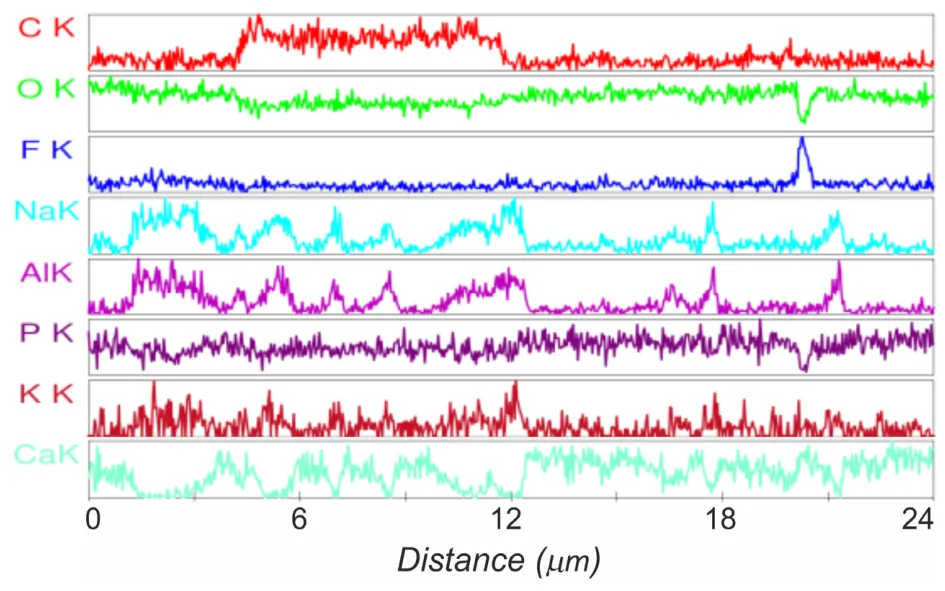
EDS analysis data of the CaFap–alumina ceramic structure for the position shown by the gray line in Figure 7.

**Figure 9:**
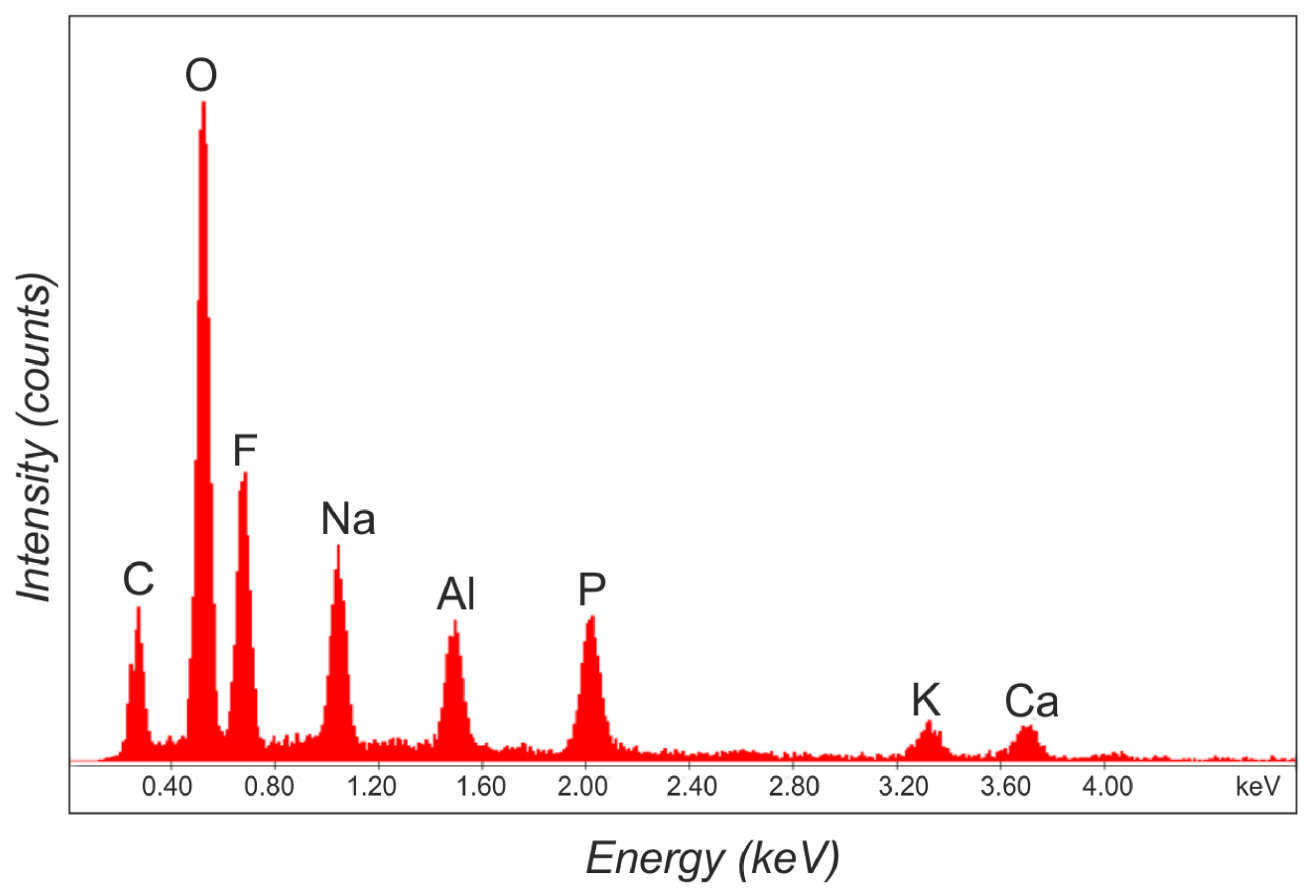
EDS spectrum corresponding to the area of the CaAlO_2_F phase in Figure 7.

**Table 1:** Atomic parameters of the new CaAlO_2_F phase (space group $\mathrm{Pm}\bar{3}m$, *a* = 4.0436(1) Å, R_Br_ = 4.78, R_f_ = 3.77).

Atom	Site	x/a	y/b	z/c	Occupancy, %
Ca	1*b*	1/2	1/2	1/2	100
Al	1*a*	0	0	0	100
M	3*d*	1/2	0	0	66.7% O + 33.3% F

## Data Availability

The raw/processed data required to reproduce these findings can be obtained from the corresponding author (grygoriy.dmytriv@lnu.edu.ua) upon reasonable request.
